# Effectiveness of Cinnamon Oil Embedded Chitosan–Gelatin Film in Inhibiting *Rhizopus oryzae*, *R. microsporus*, and *Syncephalastrum racemosum* and Controlling Rice Weevil Infestation on Paddy Rice

**DOI:** 10.3390/foods14050807

**Published:** 2025-02-26

**Authors:** Lien Thi Kim Phan, Vi Thi Mi Huynh, Nhat Minh Bui, Anh Thi Hong Le

**Affiliations:** Faculty of Food Science and Technology, Ho Chi Minh City University of Industry and Trade, 140 Le Trong Tan Street, Tay Thanh Ward, Tan Phu District, Ho Chi Minh City 70000, Vietnam; huynhthimyvi8462@gmail.com (V.T.M.H.); buinhat02@gmail.com (N.M.B.); anhlth@huit.edu.vn (A.T.H.L.)

**Keywords:** chitosan:gelatin, cinnamon oil, CO–C:G film, *R. oryzae*, *R. microsporus*, rice weevil, *S. racemosum*

## Abstract

Rice (*Oryza sativa* L.) is a staple food globally, providing a critical food for the majority of the Asian population. However, it exposes risks during post-harvest storage, threatening substantial losses in rice quality and quantity. Hence, this study developed a cinnamon oil–chitosan:gelatin film (CO–C:G film) with the parameters as the chitosan:gelatin ratio (C:G = 1:1 and 1:2), cinnamon oil (CO) contents (0.75, 1.0, 1.25, 2.5, and 5.0%) and thickness film levels (0.165, 0.183, and 0.287 mm) to inhibit three fungal species, *Rhizopus oryzae* 01, *R. microsporus* 01, and *Syncephalastrum racemosum* 01, and control rice weevil infestation on paddy rice at a variety of water activities, such as 0.71 a_w_ and 0.95 a_w_, at room temperature. The results revealed that at 0.95 a_w_, the fungal growth rate and rice weevil’s mortality were impacted significantly by all parameters of the CO–C:G film (*p* < 0.05). Especially, the CO–C:G film with 1.25% CO and C:G = 1:1 at a thickness film of 0.287 mm inhibited all observed fungi and rice weevils better than the CO–C:G film’s other parameters. The inhibition of the CO–C:G film for these fungi ranged from 66% to 72.6%. Likewise, 80–100% of rice weevils were mortal when paddy rice was treated with the CO–C:G film on the 12th or 15th day of treatment depending on the water activity of paddy rice grains. The findings of this study provide insights for researchers, agricultural experts, and the food industry, highlighting the need to establish effective and sustainable strategies for rice preservation.

## 1. Introduction

Rice (*Oryza sativa* L.) is a vital global staple crop and a primary food for the majority of the Asian population [1,2]. In post-harvest, rice is typically stored as a paddy for several months or years. However, post-harvest rice is frequently dried and stored under sub-standard technical conditions or in unsuitable instruments [3,4], particularly in regions with high humidity and elevated temperatures [5]. Such conditions significantly threaten the quality and quantity of stored paddy rice, leading to substantial losses [6]. In Southeast Asia, rice production losses during storage could reach up to 37%, with global averages around 15–16% [7]. These losses are driven by complex and variable factors, primarily stemming from interactions between abiotic conditions (e.g., temperature and moisture content) [8] and biotic factors, such as fungi, which are among the most damaging spoilage agents [9].

Several fungal species have been identified in paddy rice/rice during both pre- and post-harvest stages, including *Aspergillus* spp., *Penicillium* spp., *Fusarium* spp., *Alternaria* spp., *Mucor* spp., *Rhizopus* spp., *Trichoderma* spp., *Curvularia* spp., *Helminthosporium* spp., and *Cladosporium* spp. [6,10,11,12]. The post-harvest losses caused by fungi are an increasing concern, particularly in tropical climates, such as Vietnam, which feature high humidity and elevated temperatures [5,6].

Managing food spoilage caused by fungi is essential to minimize health risks from mycotoxin exposure and mitigate substantial economic losses. Various post-harvest technologies, including a controlled atmosphere and modified atmosphere packaging, have been employed to enhance the quality of stored paddy rice [13]. Recently, most of the rice companies have applied chemical compounds, namely “Quick—Phos 56%” (a fumigant pesticide containing 56% aluminum phosphide. Its antifungal mechanism mainly relies on phosphine (PH_3_) gas that is released during treatment. PH_3_ blocks cytochrome C oxidase in the electron transport chain, leading to the prevention of ATP synthesis and cell death, etc.) for controlling post-harvest insects and rice weevils. These treatments, typically applied through fumigation, often leave chemical residues that pose potential health risks and create trade barriers [14]. Consequently, there is a growing demand for non-chemical methods, recognized as sustainable and environmentally friendly “green storage measures”.

Essential oils (EOs) have been increasingly utilized in post-harvest management as natural alternatives to toxic chemicals due to their inherent antimicrobial compounds. The antimicrobial properties of EOs and their effectiveness against numerous post-harvest phytopathogens have been well documented over the years [15,16]. Among these, cinnamon oil (CO) stands out, having been approved by the Food and Drug Administration under the Generally Recognized as Safe category for use as a food additive [14]. CO exhibits potent antimicrobial activity even at low concentrations, primarily attributed to its high cinnamaldehyde content [17,18,19]. It is highly effective against a broad spectrum of foodborne microorganisms, including *E. coli*, *Pseudomonas aeruginosa*, *S. typhimurium*, *Bacillus subtilis*, *B. cereus*, *S. aureus*, and *L. monocytogenes* [20,21,22]. Additionally, it demonstrates strong activity against fungi and yeasts, such as *Aspergillus niger*, *A. flavus*, *Penicillium chrysogenum*, *P. expansum*, *P. citrinum*, *Mucor circinelloides*, and *Saccharomyces cerevisiae* [23]. Although the antimicrobial properties of EOs are well-documented, their practical application in food products faces significant challenges due to the strong and often unpleasant odors they produce, along with potential alterations in taste [17,18,19]. Despite these limitations, EOs have gained attention as antibacterial agents in laboratory-scale studies. Recently, several innovative approaches have been developed to overcome these constraints. These methods typically involve using smaller quantities of EOs or avoiding direct contact with food products. Examples include incorporating EOs into packaging materials, coatings, or through nanoencapsulation techniques. Among these, the incorporation of EOs into food packaging films has proven to be one of the most effective strategies for combating various pathogens [24,25]. Additionally, EOs could be encapsulated and integrated into edible or biodegradable films or coatings, enabling their controlled release into the food or the surrounding gaseous environment within the package [17,18].

In certain applications, edible films or coatings are enhanced by combining EOs with other antimicrobial agents to boost their effectiveness [26,27,28]. Another innovative approach to optimizing EO use involves encapsulating them into nanoemulsions. This technique improves the stability of the volatile components and minimizes their interactions with the food matrix [26]. For instance, refs. [29,30] demonstrated the use of EO-based nanoemulsions in washing and rinsing solutions to combat *E. coli* on fresh vegetables. However, to the best of our knowledge, no studies have yet reported a CO–chitosan:gelatin film to inhibit fungi on paddy rice and rice weevil in rice that is friendly to the environment and safe for the consumer. Therefore, the primary objective of this study was to investigate the effectiveness of a cinnamon-oil-embedded chitosan–gelatin film in inhibiting *Rhizopus oryzae*, *R. microsporus*, and *Syncephalastrum racemosum* and controlling rice weevil infestations on paddy rice under popular storage conditions.

## 2. Materials and Methods

### 2.1. Cinnamon-Oil-Embedded Chitosan–Gelatin Film

#### 2.1.1. Extraction of Cinnamon Oil

One hundred and fifty grams of cinnamon bark powder (VNTEA Trade Co., Ltd., Ho Chi Minh City, Vietnam) was combined with 600 mL of distilled water (Ho Chi Minh City University of Industry and Trade, Ho Chi Minh City, Vietnam). The mixture was placed in a distillation flask (Thermo Fisher Scientific, Cambridge, UK) and subjected to hydro distillation using a Clevenger-type apparatus (MSICO, Haryana, India). This process produced a clear, light-yellow oily layer that floated atop the aqueous distillate. The oil was carefully separated, dried at room temperature using anhydrous sodium sulfate (Merck, Darmstadt, Germany), and stored in airtight glass vials (Agilent, Santa Clara, CA, USA) wrapped in aluminum foil. The vials were maintained at 4 °C until further analysis [14].

#### 2.1.2. Gas Chromatography/Mass Spectrometry Analysis of Cinnamon Oil

The composition of CO was analyzed using gas chromatography/mass spectrometry (GC/MS) with electron ionization (EI) on a GC–MS 7890B system coupled with a 7010 Triple Quadrupole (Agilent, Stamford, CT, USA). The analysis utilized an HP–5MS capillary column (5% of phenyl–methylpolysiloxane) with dimensions of 30 m × 0.25 mm and a 0.25 µm film thickness (Agilent, Stamford, CT, USA). The temperature program was set as follows: an initial hold at 50 °C for 2 min, followed by a ramp of 5 °C/min to 200 °C, then 10 °C/min to 280 °C, which was held for 5 min. Helium was used as the carrier gas at a constant flow rate of 1 mL/min. A 1.0 mL solution (1:10 *v*/*v*, essential oil/hexane) was injected into a splitless injector with an injector temperature of 250 °C, operating in split mode (split ratio 100:1) [14].

#### 2.1.3. Cinnamon-Oil-Embedded Chitosan–Gelatin Film (CO–C:G Film)

Two percent (*w*/*v*) chitosan (Zhanyun, Shanghai, China) was transferred into a 1% (*v*/*v*) acetic acid solution (Duc Giang Chemical Group, Ha Noi, Vietnam) and stirred at 55 °C/30 min. In addition, 2% (*w*/*v*) gelatin (Xilong Scientific Co., Shantou, China) was added into distilled water and stirred at 55 °C/30 min. Next, 25% glycerol (Duc Giang Chemical Group, Ha Noi, Vietnam) (*v*/*v*) was put into the gelatin solution and stirred at 55 °C/15 min. Afterwards, the chitosan solution was mixed with the gelatin solution and stirred at 55 °C/15 min. Additionally, cinnamon oil–CO (0.75%, 1%, 1.25%, 2.5%, and 5%, *v*/*v*) (CO extracted in Section 2.1.1) was added into the chitosan–gelatin mixture and stirred at 55 °C/30 min. For the next step, 0.2% tween-80 (Xilong Scientific Co., Shantou, China) (*v*/*v*) was transferred to this mixture and then stirred at 55 °C/5 min. Finally, all the mixtures were poured into a Petri plate (90 mm, Dinlab, Göttingen, German) and dried at 40 °C/72 h until the moisture of the film was around 6% with variety of thicknesses (0.165 mm, 0.183 mm, and 0.287 mm). The CO–C:G film was cut into 1 cm × 1 cm pieces (weight: 0.15 g) and stored at 25 °C until analysis.

### 2.2. Fungal Strains and Preparation of Spore Solution

#### 2.2.1. Fungal Strains

**Figure 1:**
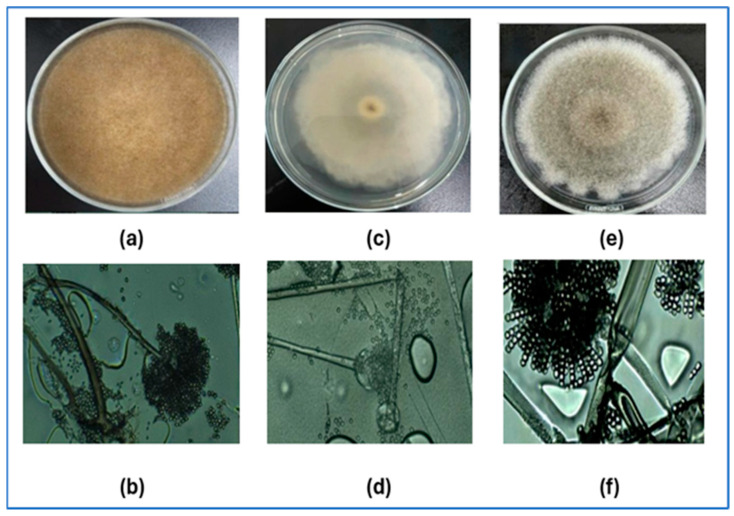
*R. oryzae*, *R. microsporus*, and *S. racemosum* on PDA (**a**,**c**,**e**); microscope (**b**,**d**,**f**).

**Figure 2:**
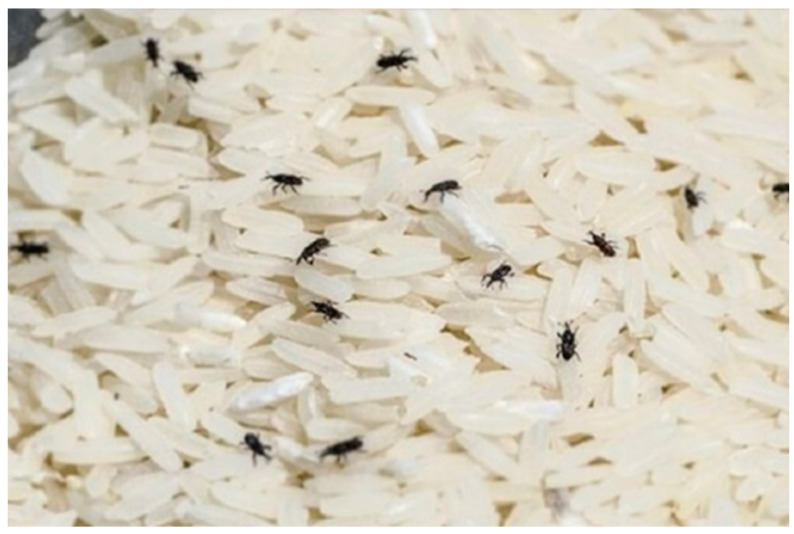
Rice weevils on rice.

*Rhizopus oryzae* 01 (RO01), *R. microsporus* 01 (RM01), and *Syncephalastrum racemosum* 01 (SR01) strains were employed (Figure 1). These strains were isolated from stored paddy rice samples collected in Mekong Delta, Vietnam in 2022 and their identification was accomplished through both macroscopic and microscopic assessments. Fungal species determination was further corroborated through a molecular analysis, utilizing ITS4 and ITS5 primers to amplify the ITS region of rRNA [6], which was conducted at the Ho Chi Minh City University of Industry and Trade in Vietnam and the 1st Base laboratory in Singapore. In addition, rice weevils were collected from citizen living in Ho Chi Minh city, Vietnam (Figure 2).

#### 2.2.2. Preparation of Fungal Spore Solution and Rice Weevils

##### Fungal Spore Solution

One hundred microliters of a spore solution (10^6^ spores/mL) for each fungus was centrally inoculated onto Potato Dextrose Agar (PDA) Petri plates (Merck, Darmstadt, Germany; Dinlab, Göttingen, Germany) and incubated at 30 °C for 7 days to facilitate spore development. Following incubation, 5 mL of a Tween 80 solution (0.1 g per 100 mL water) (Sigma–Aldrich, Waltham, MA, USA) was evenly applied to the PDA plates with fully developed fungal colonies. The mixture was transferred into sterile tubes fitted with sterile cotton plugs to remove debris. This spore extraction process was repeated twice on the same plate to maximize spore recovery. The cotton plugs were then discarded, and the tubes were centrifuged at 8500 rpm at 4 °C for 15 min. After centrifugation, the supernatant was discarded, and 20 mL of a phosphate buffered saline (PBS) solution containing Tween 80 (0.1 g Tween 80 and 1 PBS tablet per 100 mL water) was added to the spore-containing tubes. The suspension was vortexed for 30 s and centrifuged again under identical conditions. The supernatant was removed, and 20 mL of the PBS solution (1 PBS tablet per 100 mL water) was added to the tubes and vortexed to resuspend the spores. The spore concentration was quantified using a 16-cell Thoma chamber (Hirschmann, Eberstadt, Germany) under a microscope (Optika, Ponteranica, Italy). Appropriate dilutions were performed to adjust the spore concentration to 10^6^ spores/mL in PBS [14,31].

##### Rice Weevils

Rice weevils were collected from in Ho Chi Minh city and then grown on rice powder at room temperature (25 °C) in the laboratory of HUIT, Vietnam (n = 1200).

### 2.3. Infection of Fungi and Rice Weevil on Paddy Rice

The spores of RO01, RM01, and SR01 strains and rice weevils were prepared, as in Section 2.2. In addition, in order to eliminate any fungal contamination, the paddy rice samples (OM18) were irradiated with cobalt-60 gamma rays at 25 kGy at the Toan Phat Company in Long An province, Vietnam.

The initial water activity—a_w_—of paddy rice was determined using an a_w_ meter (EZ200, Freund, Tokyo, Japan). Subsequently, the a_w_ was adjusted to the desired levels (0.95 a_w_) by adding precise quantities of a glycerol solution to water (g) and to water (100 mL) according to the equations (Equation (1)). These mixtures were autoclaved at 121 °C for 15 min and subsequently cooled to room temperature. Afterwards, 18 mL of the glycerol water solution with a specific a_w_ was added to 100 g of paddy rice. In order to ensure the uniform adsorption of water, the paddy rice was equilibrated at 4 °C/48 h with regular agitation to confirm water absorption [14]. In addition, the actual a_w_ of the paddy rice was determined. Around 62 g rehydrated paddy rice samples were transferred to a glass bottle (Miso, HCM, Vietnam) (13.7 × 5.3 cm) and arranged to create a compact layer. Fifty microliters of the prepared spore solution (10^6^ spores/mL) and ten rice weevils were added at the corners of these glass bottles containing 62 g of rehydrated paddy rice, and then, a CO–C:G film (1 cm × 1 cm) that contained different chitosan:gelatin ratios (C:G = 1:1 and 1:2), cinnamon oil contents (0.75, 1.0, 1.25; 2.5, and 5.0%) and thickness levels (0.165 mm, 0.183 mm, and 0.287 mm), as mentioned in Section 2.1.3, was added at the contrast side.

Triple-replicate sets for each condition were incubated in closed plastic containers. Each container was placed in a 250 mL beaker filled with 100 mL of a glycerol–water solution, matching the a_w_ of the paddy rice to maintain a constant equilibrium relative humidity (ERH) during the incubation period [14,31,32]. The precise a_w_/glycerol combinations were initially determined by preparing various glycerol solutions in water and measuring the resulting a_w_ values. The following equation was developed [Equation (1)]

Y = −0.0022X + 1.0006  R_2_ = 0.9928 (1)

where Y is the desired water activity level (a_w_) and X is the amount of glycerol added (g/L).

### 2.4. Evaluation of Fungal Growth and Rice Weevil Mortality

Fungal growth and rice weevil mortality were assessed under distinct CO–C:G film parameters, as mentioned above, at a variety of water activity levels of paddy rice grains (0.71 and 0.95 a_w_). The growth rate of fungi was measured daily and calculated based on a square table with 100 squares (0.25 cm^2^/square) using the formula Equation (2). Fungal growth measurements continued until the fungal colony reached all the bottles. Additionally, the mean growth rate was determined by dividing the mean growth by the number of days of incubation [33,34,35]. Subsequently, the data were transformed into the percentage of inhibition of mycelial growth relative to the control treatment (0% cinnamon oil) by employing the formula of Equation (3) [36]:

The growth rate of fungi was calculated based on the following formula:

(2) $$\mu_{max}=\frac{v_{max}}{n}$$

µ_max_, mean growth rate (cm^3^·day^−1^), V_max_, Fungal growth rate volume (cm^3^), and n, the number of incubated day (day).

(3) $$I\left(Inhibition,\%\right)=\frac{\mu_{max}\;of\;control-\mu_{max}\;of\;treatment}{\mu_{max}\;of\;control}\times 100$$

The rice weevil mortality was calculated based on the following formula (Equation (4)):

(4) $$\mathrm{Rice}\;\mathrm{weevil}\;\mathrm{mortality}\;(\%)=\frac{the\;number\;of\;died\;rice\;weevils}{total\;rice\;weevils}\times 100$$

### 2.5. Statistical Analysis

In this study, each experimental condition was replicated three times. The results were expressed as the mean ± SD. Analysis of variance was performed at the significance level of *p* ≤ 0.05. The inhibitory effect was estimated using Microsoft Excel 2013 (Redmond, WA, USA).

## 3. Results and Discussions

### 3.1. Chemical Composition of Cinnamon Oil

**Figure 3:**
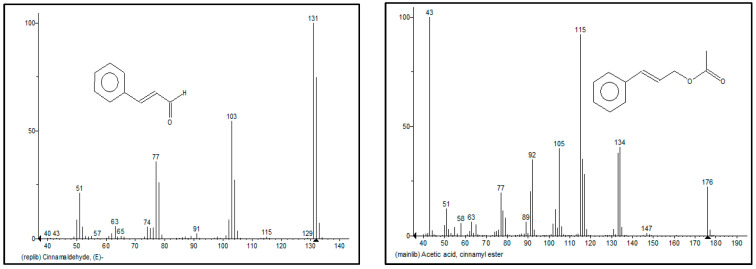
Gas chromatography–mass spectrometry spectrum of cinnamaldehyde and cinnamyl ester in cinnamon oil.

The chemical composition of cinnamon oils extracted from cinnamon bark was analyzed via GC/MS (Table 1). They consisted of alcohols (eugenol), aldehydes (cinnamaldehyde), etc., which have bioactive capacity as antimicrobials [37]. Specially, cinnamaldehyde is the major component (89.3%) containing an aldehyde group and conjugated double bond outside the ring (Figure 3). This is in agreement with the report of [38] who mentioned that cinnamaldehyde in cinnamon bark is about 65–85%. Moreover, several reports mentioned that this component could inhibit the growth of *Candida albicans*, *A. flavus*, and *Geotrichum citriaurantii* and mycotoxin production [39,40,41,42]. Additionally, ref. [14] reported that this essential oil contained around 60% of cinnamaldehyde that could inhibit not only fungal growth, including *A. flavus* and *F. proliferatum*, but also their mycotoxins, such as AFB1 and FB1.

### 3.2. Influence of the Cinnamon-Oil-Embeded Chitosan–Gelatin Films on R. oryzae 01, R. microsporus 01, and S. racemorium 01 Growth on Paddy Rice

**Figure 4:**
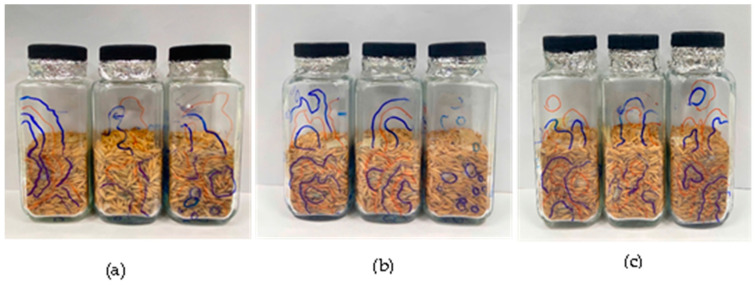
*R. oryzae* 01 (**a**), *R. microsporus* 01 (**b**), and *S. racemosum* 01 (**c**) on paddy rice at 0.95 a_w_ at room temperature, treated with a CO–C:G film (C:G = 1:1, CO: 1.25%, and thickness: 0.287 mm); the different colors of lines were marked to indicate fungal growth during research.

The CO–C:G films were developed based on chitosan:gelatin (C:G = 1:1 and 1:2), and cinnamon oil concentrations (CO: 0.75%, 1.0%, 1.25%, 2.5%, and 5.0%) with thickness levels (0.165 mm, 0.183 mm, and 0.287 mm). Subsequently, the CO–C:G films’ efficacy was evaluated based on inhibiting the growth of three fungal species (RO01, RM01, and SR01) on irradiated paddy rice at 0.71 a_w_ and 0.95 a_w_/room temperature. The results are present in Table 2 and Figure 4.

According to data present in Table 2, neither the control samples (paddy rice untreated with the CO–C:G film) nor the paddy rice treated with this film showed any fungal growth after more than 210 days of treatment at 0.71 a_w_. In contrast, fungal growth rates were impacted significantly by the CO–C:G film at 0.95 a_w_ (*p* < 0.05). However, the degree of inhibition varied depending on the fungal species and the parameters of the CO–C:G film, including the CO content, C:G ratio, and thickness level as well.

Generally, the inhibitory effect of the CO–C:G film on fungal growth increased significantly with rising CO contents in the film (*p* < 0.05); however, the degree of fungal inhibition differed among the types of fungi. Specifically, the inhibitory efficacy of the CO–C:G film against RM01 exhibited an increasing trend, ranging from 0 to 66% with a reduction in the fungal growth rate from 18.75 ± 0.00 cm^3^/day to 8.17 ± 0.05 cm^3^/day as the CO concentration increased from 0.75–5.0%. In contrast, the inhibitory ability of this film on SR01 and RO01 growth rates showed a different pattern. For these species, inhibition initially increased with CO concentrations of 0.75–1.25%, ranging as 5.0–72.6% (SR01) and 8.9–70.8% (RO01). However, at higher CO concentrations (1.25–5.0%), the inhibitory efficacy declined, with inhibition values dropping to 21.8–48.9% (SR01) and 4.9–29.1% (RO01) under the same conditions. Correspondingly, the growth rates of both fungi displayed a decreasing trend with 0.75–1.25% of CO concentrations, but this trend was reversed at higher CO levels.

When evaluating the influence of the C:G ratio in the CO–C:G film on fungal growth, the results revealed that C:G = 1:1 demonstrated stronger inhibitory effects compared to C:G = 1:2 (Table 2). For instance, the inhibition against RM01 was 55.3% with a growth rate of 8.38 ± 0.03 cm^3^/day for C:G = 1:1, whereas this value decreased to 38.2% for C:G = 1:2 with a growth rate of 11.58 ± 0.78 cm^3^/day under the same conditions. Similarly, the C:G = 1:1 was more effective in inhibiting the growth of SR01 and RO01. Indeed, the growth rate was 6.8 ± 0.12 cm^3^/day with an inhibition rate of 54.7% (SR01), while the value was 8.05 ± 0.04 cm^3^/day with an inhibition rate of 24.06% (RO01). However, at C:G = 1:2, such values for SR01 and RO01 increased to 10.85 ± 0.41 cm^3^/day and 18.75 ± 0.00 cm^3^/day with inhibition rates declining to 27.7% and 0%, respectively. The maximum inhibitory effect of the CO–C:G film on SR01 and RO01 growth was 72.6% and 70.8%, respectively, at 1.25% CO, while the maximum inhibition for RM01 was 66% at 5% of CO under the same conditions.

In addition to the influence of the aforementioned factors, fungal inhibition also depended on the thickness of the CO–C:G film. Generally, as the film thickness increased from 0.165 mm to 0.287 mm, the inhibitory efficacy on fungal growth also increased, except for RO01 at 2.5–5% of CO under all observed conditions. Specifically, at 1.25% of CO and C:G = 1:1, the fungal inhibition of the CO–C:G film increased from 37.8–70.8% (RO01), 18.9–55.3% (RM01), and 57.4–72.6% (SR01) with corresponding reductions in the mean fungal growth rates under these conditions (Table 2).

In this study, the CO–C:G film demonstrated significant inhibitory effects on the growth rates of RO01, SR01, and RM01 (*p* < 0.05). According to Phan et al., the antifungal activity of CO could be attributed to its active compounds, which interact with fungal cell membranes [14]. These interactions disrupt physiological and biochemical processes, causing cytoplasmic leakage, membrane rupture, and hyphal aggregation. Such effects lead to a significant reduction in spore production and respiration capacity, ultimately inhibiting fungal growth [43]. Additionally, CO disrupts fungal cell walls and membranes, causing cytoplasmic coagulation and damage to cellular organelles, which results in the leakage of cell contents [5].

Notably, CO contains cinnamaldehyde (89%), a key compound that inhibits ergosterol biosynthesis, thereby affecting microbial development [43]. Furthermore, Cardador et al. reported that cinnamaldehyde disrupts cell-synthesizing enzymes, damages the cell membrane, destroys mitochondria, and destabilizes the cell wall [44]. These combined effects alter fungal morphology and growth [45]. The findings in our study align with those of Xing et al. [34], who reported that CO effectively inhibits fungal growth, including *Fusarium verticillioides*, with the degree of inhibition increasing alongside the CO content [46]. Moreover, the observed increase in fungal inhibition with a higher CO content in this study is consistent with the results of [14].

The study indicated that the CO–C:G film with C:G = 1:1 inhibited the growth of RO01 and SR01 more effectively than the film with C:G = 1:2, except in the case of RM01. These findings agreed with a previous study that reported that a thyme-oil-based film with C:G = 1:1 exhibited strong antifungal activity against *Colletotrichum gloeosporioides*. Similarly, films with C:G = 1:1 incorporating cajeput oil have shown significant antibacterial properties [47]. Additional studies have also highlighted the efficacy of films with C:G = 1:1, such as those containing honey [48] or tangerine peel oil [49]. This could be attributed to the optimized interaction among chitosan, gelatin, and other film-forming components at this specific ratio, which likely improves the film’s antifungal properties when applied for preservation purposes.

According to the research data, the film’s thickness of 0.287 mm demonstrated better anti-fungal effects compared to those at 0.165 mm and 0.183 mm. This could be attributed to the CO–C:G film properties. This means that although the CO–C:G film had the same amount of CO, an increase in the thickness led to a higher CO level per unit area. Also, a thicker CO–C:G film slowed down the CO diffusion to the environment, delaying fungal growth. This result is consistent with another finding on the antibacterial activity of carboxymethyl cellulose combined with clove oil that mentioned that increasing the film thickness enhanced antibacterial effectiveness [50].

### 3.3. Influence of the CO–C:G Film on Rice Weevil on Paddy Rice

In this study, the effectiveness of the CO–C:G film in controlling rice weevils was evaluated based on mortality rates observed on the 6th, 9th, 12th, and 15th days of treatment. The control sample (paddy rice untreated with the CO–C:G film) exhibited a maximum rice weevil mortality rate of approximately 27% after 15 days of treatment (data were not mentioned in this article). In contrast, paddy rice treated with the CO–C:G film showed significantly higher weevil mortality rates. Moreover, the mortality rate was impacted by key parameters of the CO–C:G film, including its thickness, CO concentration, and C:G ratio, as well as the water activity of rice grains (0.71 and 0.95 a_w_).

**Figure 5:**
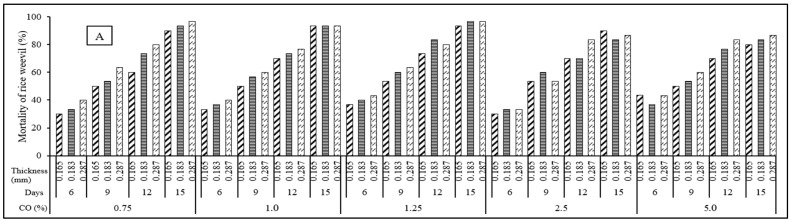

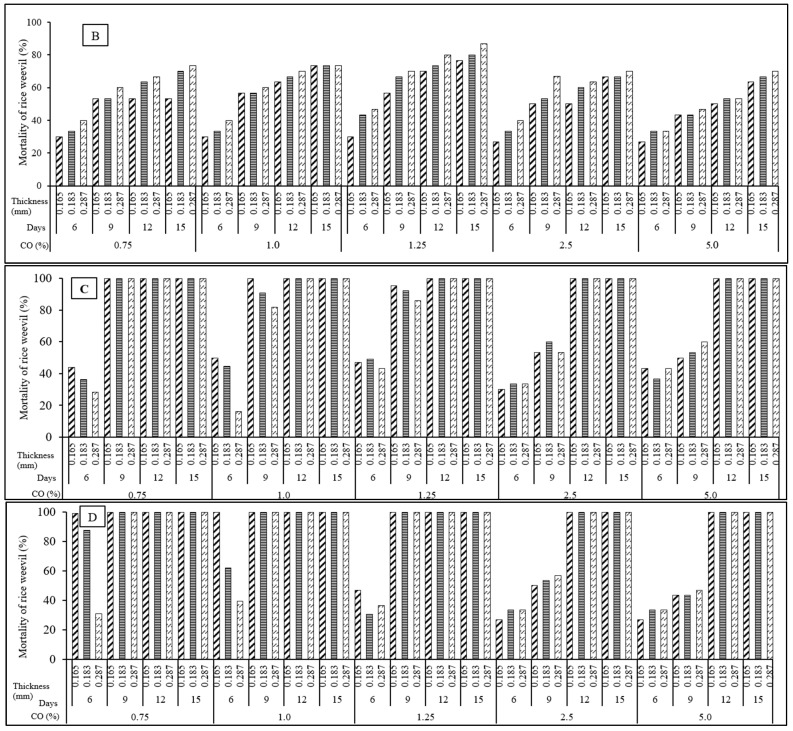
The mortality of rice weevil (%) on paddy rice at C:G = 1:1 (**A**,**C**) and C:G = 1:2 (**B**,**D**) of the CO–C:G film with different cinnamon oil levels and the thickness at 71 a_w_ (**A**,**B**) and 0.95 a_w_ (**C**,**D**).

Regarding 0.71 a_w_, the mortality rates of rice weevils are presented in Figure 5A,B. According to the data, as the CO levels increased from 0.75 to 1.25%, the mortality rates of rice weevils also increased. However, when the CO contents rose further ranging from 1.25 to 5%, the mortality rates tended to decrease at all observed conditions. In addition to the essential oil concentration, the thickness of the CO–C:G film significantly influenced rice weevil mortality. As the film thickness increased from 0.165 mm to 0.287 mm, the mortality rate also rose (Figure 5A,B). For instance, the mortality rate increased from 50 to 63% and from 63 to 73% at the same conditions on the 9th and 12th days of treatment, respectively. In addition, the C:G ratio impacted the mortality rate of rice weevils, with higher mortality observed at C:G = 1:1 compared to C:G = 1:2 (Figure 5A,B). Specifically, the mortality rates were 63–70% and 70–76% (C:G = 1:2), whereas these values increased to 70–76% and 80–83% (C:G = 1:1) at CO concentrations of 1% and 1.25% on the 12th day of treatment, respectively. Overall, the mortality rates ranged from 33 to 96.7% on the 6–15th days of treatment.

Similar to the observations at 0.71 a_w_, the mortality rate of rice weevils was influenced by the parameters of the CO–C:G film (Figure 5C,D) at 0.95 a_w_. Indeed, the results showed that increasing CO levels of this film from 0.75 to 1.25% resulted in a rise in rice weevil mortality rates, but the values tended to decrease at further CO contents, which was also discovered at 0.71 a_w_. For example, the mortality rate was 28–49% (CO: 0.75–1.25), whereas this value was reduced to 37% (CO: 1.25–5%) at C:G = 1:1 on the 6th day of treatment (Figure 5C). Overall, all rice weevils were mortal after 9 days of treatment (CO: 0.75–1.25%); however, these insects were completely controlled after 12 days of treatment at higher CO contents, regardless of the other CO–C:G film parameters. In addition, C:G = 1:2 of this film was more effective on rice weevil mortality compared to C:G = 1:1, which significantly differed from the results observed at 0.71 a_w_. Specifically, the rice weevil mortality was 16–50% (C:G = 1:1) and 33–100% (C:G = 1:2) at 1% of CO on the 6th day treatment. Furthermore, the rice weevil mortality rate increased due to a decrease in the CO–C:G film thickness at 0.75–1.25% of CO. However, there was no clear difference in rice weevil mortality as the CO–C:G film thickness was increased at further CO contents.

In this study, the mortality rate of rice weevils increased, which is associated with CO’s components and properties, particularly cinnamaldehyde, a highly toxic compound. According to [51,52,53], cinnamaldehyde indicates high toxicity against larvae and insects at different developmental stages. This compound penetrates the insect’s body and acts as a pesticide or insect repellent, disrupting certain biological processes, such as the growth rate [51].

Additionally, other reports have indicated that mono-terpenoids could cause insect mortality by inhibiting the activity of the enzyme acetylcholine esterase (AChE) [54], which hydrolyzes ester bonds. AChE plays a critical role in regulating the neurotransmitter acetylcholine (ACh). When AChE is inhibited, ACh accumulates excessively, leading to neural dysfunction and ultimately death [55]. Thus, the cinnamaldehyde present in cinnamon oil proves to be highly effective in controlling weevils, with higher mortality rates observed as the CO concentration and thickness of the CO–C:G film increase. Furthermore, the data indicated that rice weevils’ mortality rate increased significantly after the 6–15th day of treatment. Although on 6th day of treatment, the CO content could not have been strong enough to reduce all rice weevils, on further days, as more CO could be released, this led to a sharp rise in the mortality rate of rice weevils from approximately 30 to 100% on the 6–15th day of treatment. This demonstrates that CO effectively eliminates rice weevils, leading to a fall in survival. Several studies have reported similar findings, showing that higher CO concentrations and prolonged exposure times enhance the efficacy of essential-oil-based films. For instance, the mortality rates of rice weevils were 80% within one day at 1.2 mg/cm^2^ and 95.3% after four days [52,56], whereas 99.6% of rice weevil mortality was estimated on the 10th day treatment at 30 µL/500 cm^3^ against adult weevils [53]. Based on the data, the mortality rate of weevils depended on the parameters of the films. This finding was consistent with the results reported by [52]. According to their study, increasing the concentration of CO ranging from 0.6 to 0.8 mg/cm^2^ raised the weevil mortality rate from 45.4 to 56.5%. Another study similarly demonstrated that higher CO levels significantly increased weevil mortality, reaching up to 99.55% (30 μL/500 cm^2^), and inhibited the emergence of the next generation to be 98.81% [53].

## 4. Conclusions

The growth rates of fungi and rice weevils were significantly influenced by the CO–C:G film under all conditions, including the film thickness, CO concentration, and C:G ratio at 0.95 a_w_ (*p* < 0.05). Notably, the CO–C:G film containing 1.25% CO with C:G = 1:1 and a thickness of 0.287 mm exhibited the most effective inhibitory effect on fungal growth and rice weevils compared to the other parameters. Furthermore, the treatment of rice with the CO–C:G film achieved 80–100% of rice weevil mortality on the 12th or 15th day, depending on the water activity of the grains. These findings suggest that CO–C:G films revealed significant potential for controlling fungal contamination and insects in stored paddy rice. Further research is warranted to evaluate the industrial applicability of CO–C:G films in paddy rice storage for mitigating contamination by mycotoxin-producing fungi, such as *Aspergillus flavus*. Collaborative efforts among researchers, agricultural experts, and the food industry are crucial to developing effective and sustainable strategies for rice preservation.

## Figures and Tables

**Table 1:** Chemical composition of cinnamon oil.

Compounds	Retention Time (min)	Amount (%)	MS Compatibility
α-Pinene	10.545	0.34	91.5
o-Cymene	13.233	2.14	94.4
D-Limonene	13.363	0.41	90.5
Eucalyptol	13.466	0.08	91.5
Linalool	15.411	1.09	81.7
α-Terpineol	18.412	0.05	90.7
Cinnamaldehyde	20.441	89.29	90.6
Eugenol	22.628	0.37	93.5
Caryophyllene	24.417	1.86	92.8
Cinnamyl acetate	24.788	4.38	91.9

MS: mass spectrometry.

**Table 2:** Fungal growth rate (Mean ± SD—cm^3^/day and fungal inhibition (I%) on paddy rice treated with CO–C:G film.

Water Activity (a_w_)	Fungi	C:G	Thickness (mm)	Cinnamon Oil—CO (%)											
				0.75		1.0		1.25		Control 1 (cm^3^/Day)	2.5		5.0		Control 2 (cm^3^/Day)
				Mean ± SD (cm^3^/Day)	I (%)	Mean ± SD (cm^3^/Day)	I (%)	Mean ± SD (cm^3^/Day)	I (%)		Mean ± SD (cm^3^/Day)	I (%)	Mean ± SD (cm^3^/Day)	I (%)	
0.71	*RO01*; *RM01* and *SR01*	1:1 and 1:2	0.165; 0.183 and 0.287	–	–	–	–	–	–	–	–	–	–	–	–
0.95	*R. oryzae 01*	1:1	0.165	8.94 ^aA^ ± 0.04	15.67	8.05 ^bA^ ± 0.04	24.06	6.57 ^cA^ ± 0.02	37.83	10.00 ± 0.00	6.40 ^bB^ ± 0.33	44.51	8.12 ^aA^ ± 0.54	29.62	11.54 ± 0.00
			0.183	7.78 ^aB^ ± 0.04	26.56	7.03 ^bB^ ± 0.03	33.56	5.63 ^cB^ ± 0.03	46.67		5.33 ^aC^ ± 0.32	53.78	4.08 ^bB^ ± 0.27	64.62	
			0.287	7.48 ^aC^ ± 0.02	29.33	6.91 ^bC^ ± 0.05	34.67	3.04 ^cC^ ± 0.03	70.83		8.02 ^aA^ ± 0.10	30.51	8.18 ^aA^ ± 0.17	29.12	
		1:2	0.165	9.67 ^aA^ ± 0.03	8.89	8.30 ^bA^ ± 0.02	21.67	7.46 ^cA^ ± 0.04	29.50		8.25 ^bB^ ± 0.27	28.51	10.30 ^aB^ ± 0.30	10.73	
			0.183	9.02 ^aB^ ± 0.02	15.00	7.77 ^bB^ ± 0.04	26.78	6.06 ^cB^ ± 0.03	42.61		7.65 ^bB^ ± 0.10	33.73	8.69 ^aC^ ± 0.16	24.73	
			0.287	8.23 ^aC^ ± 0.03	22.33	7.18 ^bC^ ± 0.02	32.28	4.57 ^cC^ ± 0.02	56.50		11.29 ^aA^ ± 0.35	2.18	10.97 ^aA^ ± 0.13	4.90	
	*R. microsporus 01*	1:1	0.165	16.10 ^aA^ ± 0.72	14.11	15.51 ^aA^ ± 0.08	17.28	15.21 ^aA^ ± 0.48	18.89	24.02 ± 0.02	10.83 ^aA^ ± 0.01	54.90	10.87 ^bA^ ± 0.02	55.14	24.02 ± 0.02
			0.183	14.23 ^aB^ ± 0.50	24.11	13.74 ^aA^ ± 0.69	26.72	13.46 ^aAB^ ± 0.43	28.22		9.29 ^aB^ ± 0.03	61.33	8.76 ^bB^ ± 0.02	63.55	
			0.287	13.02 ^aB^ ± 0.08	30.58	10.98 ^aB^ ± 0.45	41.42	8.38 ^aB^ ± 0.03	55.33		9.11 ^aC^ ± 0.06	62.08	8.17 ^bC^ ± 0.05	65.97	
		1:2	0.165	18.75 ^aA^ ± 0.00	0.00	18.75 ^aA^ ± 0.00	0.00	16.14 ^bA^ ± 0.68	13.94		11.54 ^aA^ ± 0.00	51.96	11.15 ^bA^ ± 0.03	53.56	
			0.183	16.98 ^aB^ ± 0.87	9.44	16.75 ^aB^ ± 0.53	10.67	15.49 ^aA^ ± 0.16	17.39		10.94 ^aB^ ± 0.06	54.45	9.48 ^bB^ ± 0.03	60.53	
			0.287	14.56 ^aC^ ± 0.08	2.36	14.18 ^aC^ ± 0.54	24.39	11.58 ^bB^ ± 0.78	38.22		10.47 ^aC^ ± 0.06	56.39	9.29 ^bC^ ± 0.05	61.33	
	*S. racemosum 01*	1:1	0.165	8.23 ^aA^ ± 0.13	45.11	6.8 ^bA^ ± 0.12	54.67	6.38 ^bA^ ± 0.2	57.44	15.00 ± 0.00	7.04 ^aA^ ± 0.21	29.56	6.31 ^bA^ ± 0.23	36.94	10.00 ± 0.00
			0.183	7.09 ^aB^ ± 0.06	52.72	6.23 ^bB^ ± 0.09	58.44	5.04 ^cB^ ± 0.43	66.39		6.7 ^aAB^ ± 0.07	33.00	5.39 ^bAB^ ± 0.46	46.11	
			0.287	6.41 ^aC^ ± 0.13	57.28	5.74 ^bC^ ± 0.18	61.72	4.11 ^cC^ ± 0.17	72.61		6.32 ^aB^ ± 0.08	36.83	5.11 ^bB^ ± 0.03	48.89	
		1:2	0.165	14.25 ^aA^ ± 0.19	5.00	10.85 ^bA^ ± 0.41	27.67	9.05 ^cA^ ± 0.16	39.67		9.56 ^aA^ ± 0.05	4.39	7.82 ^bA^ ± 0.23	21.78	
			0.183	13.38 ^aB^ ± 0.21	10.83	9.51 ^bB^ ± 0.21	36.61	8.38 ^cB^ ± 0.26	44.17		9.32 ^aB^ ± 0.02	6.72	7.63 ^bA^ ± 0.06	23.72	
			0.287	12.64 ^aC^ ± 0.08	15.72	8.46 ^bC^ ± 0.06	43.61	7.63 ^cC^ ± 0.09	49.17		9.05 ^aC^ ± 0.01	9.44	7.14 ^bB^ ± 0.13	28.56	

(–): no growth; Control 1 was used to calculate I% for 0.75%, 1.0%, and 1.25% of CO, while Control 2 was applied to calculate I% for 2.5% and 5.0% of CO; I%: inhibition, was estimated based on [Equation (3)], C:G: chitosan:gelatin, mean ± SD: mean growth rate; Letters ^a–c^ express significant differences among COs (%) at the same C:G ratio and thickness of the CO–C:G film, and letters ^A–C^ express significant differences among thicknesses at the same C:G and CO content.

## Data Availability

The raw data supporting the conclusions of this article will be made available by the authors on request.
